# Intraframe motion correction for raster-scanned adaptive optics images using strip-based cross-correlation lag biases

**DOI:** 10.1371/journal.pone.0206052

**Published:** 2018-10-25

**Authors:** Mehdi Azimipour, Robert J. Zawadzki, Iwona Gorczynska, Justin Migacz, John S. Werner, Ravi S. Jonnal

**Affiliations:** 1 Vision Science and Advanced Retinal Imaging Laboratory (VSRI), Department of Ophthalmology and Vision Science, UC Davis Eye Center, Sacramento, CA, United States of America; 2 Department of Physics, Astronomy and Informatics, Nicolaus Copernicus University, Torun, Poland; University of Melbourne, AUSTRALIA

## Abstract

In retinal raster imaging modalities, fixational eye movements manifest as image warp, where the relative positions of the beam and retina change during the acquisition of single frames. To remove warp artifacts, strip-based registration methods–in which fast-axis strips from target images are registered to a reference frame–have been applied in adaptive optics (AO) scanning light ophthalmoscopy (SLO) and optical coherence tomography (OCT). This approach has enabled object tracking and frame averaging, and methods have been described to automatically select reference frames with minimal motion. However, inconspicuous motion artifacts may persist in reference frames and propagate themselves throughout the processes of registration, tracking, and averaging. Here we test a previously proposed method for removing movement artifacts in reference frames, using biases in stripwise cross-correlation statistics. We applied the method to synthetic retinal images with simulated eye motion artifacts as well as real AO-SLO images of the cone mosaic and volumetric AO-OCT images, both affected by eye motion. In the case of synthetic images, the method was validated by direct comparison with motion-free versions of the images. In the case of real AO images, performance was validated by comparing the correlation of uncorrected images with that of corrected images, by quantifying the effect of motion artifacts on the image power spectra, and by qualitative examination of AO-OCT B-scans and *en face* projections. In all cases, the proposed method reduced motion artifacts and produced more faithful images of the retina.

## Introduction

In adaptive optics scanning laser ophthalmoscopy (AO-SLO) and scanning adaptive optics optical coherence tomography (AO-OCT), the imaging beam is scanned across the retina in a two-dimensional raster pattern, wherein different parts of the retinal patch are imaged at different times. Because of this, lateral eye movements manifest in the resulting image as compressions or expansions (when the eye moves parallel to the slow scanner) and shear (when the eye moves parallel to the fast scanner). In addition to these artifacts, volumetric AO-OCT images suffer from axial shear due to axial eye movements. We refer to these artifacts collectively as *image warp*.

Rigid-body (RB) registration [1] is suitable for registration of AO-flood images [2–7], because all portions of the image are acquired at once. In raster scanning systems such as AO-SLO and AO-OCT, RB registration is not suitable. Stevenson and Roorda proposed an approach [8, 9] in which single lines (or small groups of lines) in the AO-SLO image are treated as rigid bodies, while the frame as a whole is not. First, a reference frame relatively free from motion artifacts is selected. Next, the images to be registered are divided into target strips (oriented along the axis of the fast/resonant scanner), and those strips are registered to the reference image, using cross-correlation or alternative statistical approaches. In AO-OCT volumetric images, the same approach can be applied after segmentation and projection of a high-contrast layer such as the photoreceptor mosaic [10, 11], or in conjunction with simultaneous AO-SLO imaging [12]. Once volumes have thus been registered in the lateral dimensions, the cross-sectional B-scan images may be cut into vertical strips and aligned to a reference B-scan in an exactly analogous approach.

Strip-based registration has enabled a variety of important findings, such as *in vivo* visualization of RPE cells [13], the complete rod mosaic [14], and postreceptoral retinal neurons [15]. Applied to AO-OCT images it has permitted tracking of outer segment renewal [11], disc shedding [16], and volumetric visualization of RPE cells [17] and retinal ganglion cells [18]. It has also been applied to OCT angiography images in order to improve SNR of angiograms [19, 20].

In all of these applications, selection of a good reference frame is a necessary first step. Because the living human eye is always moving, motion artifacts are present in all of the frames. Typically frames with minimal visible motion artifacts are selected, and approaches for automating the selection of a reference frame have been developed [21]. Nevertheless, in raster-scanned imaging modalities, eye movement artifacts are presumed to be present in every frame, whether or not they are detectable via visual inspection or automated methods, with the impact of these artifacts inversely related to the speed of image acquisition.

Statistical methods for correcting reference frame artifacts have been proposed [8, 22], and recently an approach based upon those proposals was validated using AO flood illumination images of the retina artificially warped using simulated eye movements [23]. The latter study examined two approaches for reference intraframe motion correction. In the “simple” approach, strips from corresponding parts of the reference and target frame are registered, and the displacements are averaged across target frames in order to infer intraframe motion in the reference frame. In the “combined” approach, the simple approach is combined with “robust registration”, in which strips of the reference image are registered to strips in the target images containing corresponding tissue. The latter approach was implemented using RB registration of targets to the reference initially, so that the location of strips of corresponding tissue can be predicted.

Here we implement the Bedggood and Metha algorithm using registration of reference strips to whole target images. Because we are interested in dewarping AO-OCT in addition to AO-SLO images, the former of which are acquired at roughly 1/10 the rate of the latter, RB registration techniques are unreliable even for approximate bulk correction of eye movements. The method proposed here obviates an RB registration step. We validate the method 1) directly with simulated motion-warped AO-SLO images, where the coordinates of photoreceptors are precisely known, and 2) indirectly with AO-SLO and AO-OCT images acquired on human subjects.

## Materials and methods

### Simulation of cone mosaic and eye movements

In order to test the lag-bias reconstruction algorithm with the benefit of a ground truth for comparison, we developed models of the cone photoreceptor mosaic and fixational eye movements. All simulation of images and analysis of simulated and real images was performed using the Python scientific stack (Python/Numpy/Scipy/Matplotlib). Sample data, as well as software and documentation for generating simulated images and for performing registration and intraframe motion correction on simulated and real images are available at https://github.com/rjonnal/intraframe_motion_correction.

**Fig 1:**
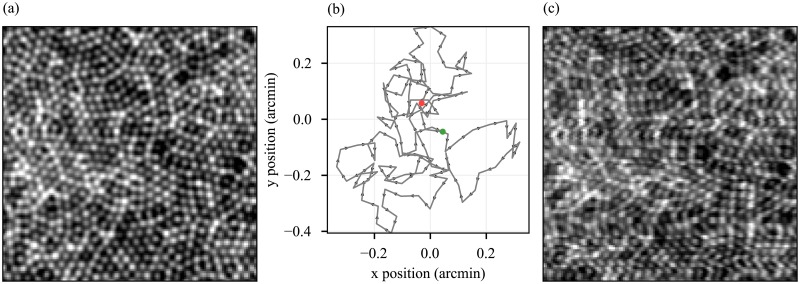
Simulation of AO-SLO image with eye motion artifacts. (a) A cone mosaic phantom. (b) A simulated eye movement traced, using a self-avoiding walk model of fixational eye movements. Units on both axes are visual angle in arcmin. (c) Simulated effect of eye movements in (b) on raster-scanned image of (a). Horizontal and vertical components of eye movements are visible in shearing and compression or expansion of image features, respectively.

The cone mosaic was simulated using a particle system, in which a circular space corresponding to a known retinal radius was seeded at random with a set of points corresponding to the number of cones expected to lie in the area. These points were subjected to a field consisting of inverse square repulsion from other points combined with inverse square attraction to the center of the field, and their positions and the total field iteratively recomputed. When the average motion per iteration was sufficiently small, the points were randomly assigned intensities according to intensity distributions from real retinal images, and then convolved with a two-dimensional Gaussian function. The key parameters–strengths of the local repulsive and global attractive fields–were adjusted until the eccentricity-density curve matched well-known histological examples [24]. An example of a synthetic image is shown in Fig 1(a).

In order to simulate eye movements, we implemented a self-avoiding walk model previously shown to predict eye movements accurately [25]. The parameters of the model were adjusted such that the amplitude, maximum speed, and mean speed of fixational drift fell within ranges reported in the literature [26]. An example of eye movement trace, with a duration of 33 ms (equal to the acquisition time for a single SLO frame), is shown in Fig 1(b).

To generate a series of simulated motion-affected cone mosaic images, we used the eye movement model to generate a movement trace of duration equal to the series acquisition time. Next, we virtually raster-scanned the simulated mosaic, using scanning parameters from our AO-SLO, while moving the simulated mosaic according to the movement trace. An example of the resulting motion-affected images is shown in Fig 1(c). The full series of frames contained images with similar, but uncorrelated, movement artifacts.

### Imaging systems

**Fig 2:**
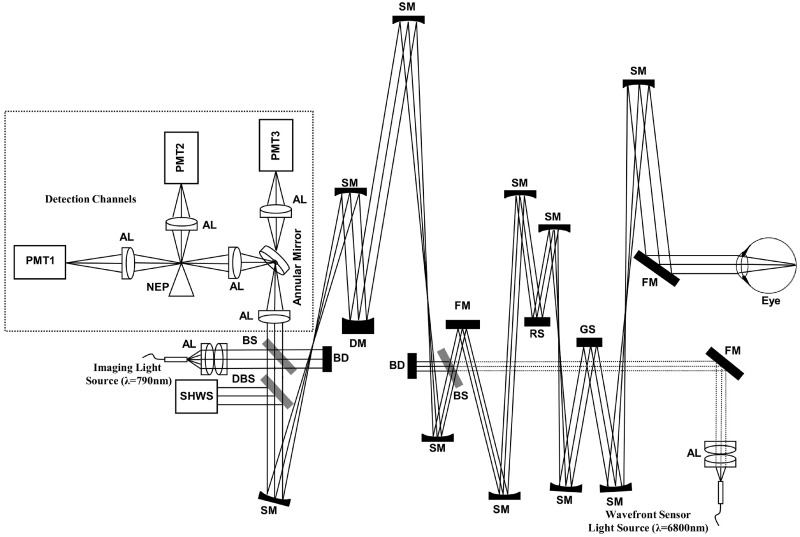
Schematic of the multi-modal AO-SLO system. DM, deformable mirror; SHWS, Shack–Hartmann wavefront sensor; AL, achromatic lens; PMT, photomultiplier tube; SM, spherical mirror; FM, flat mirror; BS, beam splitter; DBS, Dichroic beam splitter; KEP, knife edge prism; RS, resonant (x) scanner; GS, galvanometer (y) scanner; BD, beam dump.

In order to obtain real images of the cone mosaic, we constructed an adaptive optics scanning laser ophthalmoscope (AO-SLO), an optical imaging modality which provides retinal images with cellular resolution [27]. A schematic of the AO-SLO system is shown in Fig 2 [12]. AO provided diffraction-limited imaging over a dilated 6.75 *mm* pupil, by measuring and correcting ocular aberrations in closed-loop, using a wavefront sensor (20x20 lenslet array, Northrop-Grumman; 1M60 CCD camera, Dalsa) and deformable mirror (DM-97-15, ALPAO SAS), respectively, at a rate of 15 Hz. The AO beacon was a 680 *nm* superluminescent diode (Superlum Ltd, Moscow, Russia), with power measured at the cornea of 20 *μW*.

SLO images were acquired by focusing light from a separate 780 *nm* SLD (Superlum Ltd) on the retina, raster scanning the beam over a 2° patch of retina, and detecting the back-scattered light. The power of the imaging SLD was 200 *μW*, measured at the cornea. Diffraction-limited imaging at this wavelength provides a lateral resolution of 2.4 *μm* in the eye (3.4 *μm* in air, validated using a USAF target).

The detection channel was modified by replacing the confocal pinhole with a custom-made annular reflective mirror to implement confocal, split-detector [28, 29] and dark-field [30, 31] modalities. The inner diameter of the annular reflective mirror used for imaging was set to 3 Airy disk diameters while the outer diameter was 500 *μm*. The reflected beam was split equally using a knife-edge-prism (MRAK25-P01, Thorlabs, NJ) and the two halves of the signal were detected separately (PMT1 and PMT2 in Fig 2) and subtracted or added together to implement split-detector and confocal images, respectively. The split-detector implementation in this work is similar to Scoles et al.’s [29], which measured largely non-confocal signal in order to improve spatial resolution. The dark field imaging modality was obtained by rejecting the confocal signal and detecting multiply-scattered light (PMT3 in Fig 2).

AO-OCT images were acquired using our custom AO-OCT system, described in detail elsewhere [32].

Two subjects, free of known retinal disease, were imaged after obtaining informed consent. Each subject’s eye was dilated and cyclopleged by instilling topical drops of 2.5% phenylephrine and 1% tropicamide. To reduce motions during retinal imaging, a bite-bar and a forehead-rest were employed and assembled on a motorized X-Y-Z translation stage to precisely adjust the position of the subject’s eye pupil in the center of the imaging system entrance pupil. During imaging, a calibrated fixation target was employed to position the eye at specified retinal locations as well as to reduce eye movements. All procedures were in accordance with the tenets of the Declaration of Helsinki and were approved by the University of California, Davis Institutional Review Board.

### Strip-based image registration of AO-SLO images

#### Selection of a reference frame

**Fig 3:**
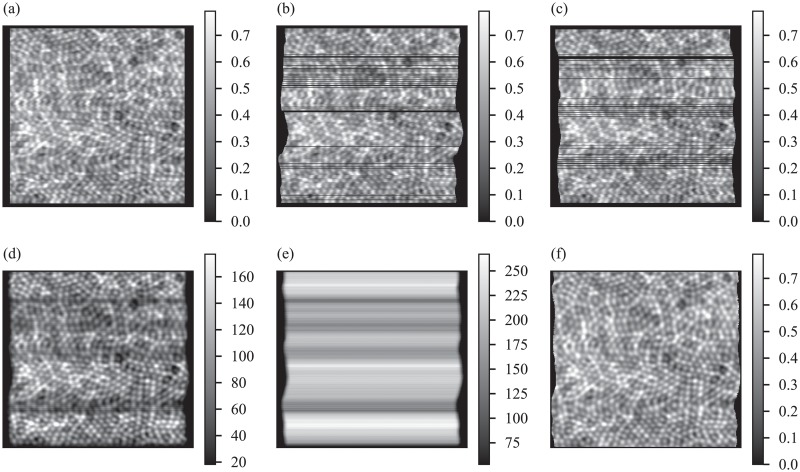
Strip-based registration of synthetic AO-SLO images. Registration begins with selection of a reference frame (a) from the series of 200 images to be registered. Next, each target image in the series is partitioned into a series of horizontal strips. Using two-dimensional cross-correlation, these strips are registered and aligned with the reference frame. Two example targets are shown in (b) and (c), after alignment to the reference. Displacements between position of a strip in the reference image and the aligned target are referred to as *lags*. Iterating through the target images, a running sum of the aligned images is stored, shown in (d). Because the retina is moving randomly, some parts of it are imaged more than other parts. As such, while calculating the sum of aligned targets, it is necessary to keep a counter image (e), which stores the number of strips contributing to any part of the sum. Once all of the target images have been partitioned, registered, aligned, and added, the sum image (d) is divided by the counter image (e) to produce a registered average (f). Artifacts of eye motion are clearly visible in the registered average (f). These artifacts are also manifest in the counter image (e), with horizontal motion causing lateral warp in the counter and vertical motion causing variations in its amplitude. Units in (e) are number of strips, and the units in the other images are arbitrary measures of intensity.

Our procedure for registering AO-SLO images (both synthetic and real) was adapted from previously published approaches [1, 8, 9, 13]. First, from a series of images, a single frame was selected as a *reference* frame *I*_Γ_ (Fig 3(a)). The process of reference frame selection was semi-automated, using the expected spatial frequency of the cones *F*_*c*_ as a criterion [24]. In regions of the cone mosaic with broad spatial frequency spectra, such as near the fovea, the expected frequency of the center of the image was used, with the possible result that motion artifacts were present in other parts of the image, but we did not observe this problem in practice. Each image in the series was cut into strips of 32 pixels. These strips were zero-padded, discrete Fourier transformed (DFT), and the square modulus was calculated and radially averaged. The radially averaged power spectra from all strips in a given image were averaged, and from the resulting spectrum the total power in the interval of [0.95 *F*_*c*_, 1.05 *F*_*c*_] was calculated and assigned as a figure of merit for that frame. The frame with the highest power in the cone frequency range was selected as a reference frame *I*_Γ_. The reference frame was then oversampled by a factor *k* (using discrete Fourier transformation (DFT), zero-padding, and inverse DFT).

#### Registration of image series to reference frame

Once *I*_Γ_ was selected, the series of *F* images to be registered were oversampled by the same factor *k*. We refer to these images as *target* images, numbered *I*_0_, *I*_1_, …*I*_*F*−1_. Each target image consisted of *R* rows. In our system, the fast resonant scanner performs the horizontal scans, while the slow galvo scanner performs the vertical scans. As such, the strips were oriented horizontally, and indexed to the rows of the image. From each target image *I*_*f*_, a series of *R* overlapping strips was generated, numbered *r* = 0, *r* = 1, …*r* = *R* − 1. Analogously, we number columns of pixels in the image *c* = 0, *c* = 1, … *c* = *C* − 1. Practically, each strip image was generated by multiplying the target image by a rectangular window with width *W* centered about a row *r* of the target. For a given target image, the *r*^*th*^ window was a binary image with rows *p* set to one if sufficiently close to *r*, and zero otherwise:

$$\begin{array}{c} \omega_{r}\left(p\right)=\{\begin{array}{cc} 1, & \text{if}\;|p-r|\leq W/2\\ 0, & \text{otherwise} \end{array} \end{array}$$ (1)

Each strip was registered to *I*_Γ_ using normalized cross-correlation, implemented with DFT (F). For a strip centered about row *r* in frame *f*, the coordinates of the most closely matching region in *I*_Γ_ are given by:

$$\begin{array}{c} s_{x}\left(f,r\right),s_{y}\left(f,r\right)=\text{argmax}\left(F^{-1}\left[F\left(I_{\Gamma }\right)\cdot F{\left(I_{f}\cdot \omega_{r}\right)}^{*}\right]\right) \end{array}$$ (2)

In the present work, a strip size *W* of 9 pixels was used for the AO-SLO images (synthetic and real), and 7 pixels for AO-OCT images. Similarly, an oversampling factor of three was chosen for *k* as a compromise between sub-pixel precision and computational efficiency. In order to visualize registered single frames, the maps *s*_*x*_ and *s*_*y*_ were used to position individual lines into the coordinate space of the reference image. Two examples are shown in Fig 3(b) and 3(c). In this step the image into which the lines are inserted was expanded to accommodate target lines that had shifted beyond the edges of the reference image, using the maximum and minimum values given by the maps *s*_*x*_ and *s*_*y*_. In order to produce a registered average of all frames in the series, two expanded images are created: a sum image to which strips are added and a counter image to which 1 is added wherever a strip has been added in the sum image. Examples of sum and counter images are shown in Fig 3(d) and 3(e), respectively. Once all of the strips have been added in this way, the sum image is divided by the counter image, after adding machine epsilon to the latter to avoid zero-division errors. In visualizing registered frames or creating a registered average, images were oversampled by *k* beforehand, which produces an average image with sub-pixel registration precision.

#### Registration of AO-OCT series to reference volume

Lateral registration of AO-OCT volumes requires initially an axial projection of the volume over a high-contrast region such as the IS/OS and COST layers. These projections can be strip-registered just as AO-SLO images.

Axial registration of AO-OCT volumes proceeds in a manner analogous to lateral registration. First, a lateral projection of the volume, along the axis of the fast scanner, is generated. The axial dimension of this projection is analogous to the resonant scanner dimension of AO-SLO images, and projections of corresponding axial strips from the target volumes are registered to the reference projection.

### Motion correction using lag biases

#### Reconstructing eye motion traces from strip registration coordinates

The key idea of the lag bias approach is that if eye movements are uncorrelated among the target images, then the difference between lags of adjacent strips, averaged over target images, will approach zero as the number of target images increases. This is the central motivation of similar, previously published approaches as well [8, 23]. Deviations of these neighbor lag differences from zero are indications of movement in the reference frame. The process of measuring these biases is a kind of inversion of strip-based registration. Instead of dividing target images into strips and registering them to the reference image, we divide the reference image into strips and register each strip to each of the whole target images. The average position of each strip among the target images is then used as an estimate of the eye’s position during acquisition of the strip in the reference image. Using the computed eye trace, it is then possible to dewarp the reference frame and recover an image of the retina relatively unaffected by eye movement artifacts. We will refer to the x and y coordinates of strip *r* in frame *f* as $\hat{s_{x}}$ and $\hat{s_{y}}$, respectively:

$$\begin{array}{c} \hat{s_{x}}\left(f,r\right),\hat{s_{y}}\left(f,r\right)=\text{argmax}\left(F^{-1}\left[F\left(I_{\Gamma }\cdot \omega_{r}\right)\cdot F{\left(I_{f}\right)}^{*}\right]\right) \end{array}$$ (3)

**Fig 4:**
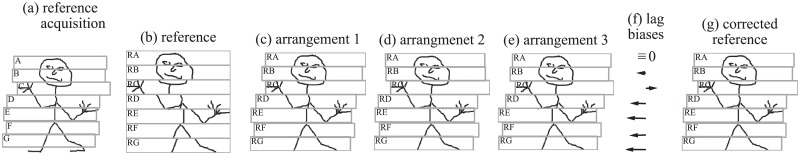
Motion correction using lag biases. The reference image (left) can be considered as a discrete set of image strips. During motion correction, the reference image is cut into strips, which are then registered, by cross-correlation, to each of the target images. Because eye motion is uncorrelated among the target images, the average position of a given reference strip is an indication of the eye’s position during its acquisition. The lag biases were used to estimate eye movements during acquisition of the reference frames, and then computationally removed from the reference frames using two-dimensional linear interpolation.

These strip lags can be visualized in Fig 4, as the registered arrangements of reference strips RA—RG on three target images. Once we have assembled from strip 1 to 2 (or 2 to 3 or 3 to 4) in targets A, B, and C, whose strips have been registered and aligned to the reference frame. Once the reference strips are registered on each target, we remove outliers (see below) and compute the lag biases ${\hat{x}}_{t}$ and ${\hat{y}}_{t}$ by averaging over frames:

$${\hat{x}}_{t}=\frac{\sum_{f=0}^{F-1}\hat{s_{x}}\left(f,r\right)}{F}$$ (4)

$${\hat{y}}_{t}=\frac{\sum_{f=0}^{F-1}\hat{s_{y}}\left(f,r\right)}{F}$$ (5)

We hypothesize that the resulting average lags ${\hat{x}}_{t}$ and ${\hat{y}}_{t}$ are estimates of the eye’s movement during acquisition of the reference frame, in a space oversampled by the factor *k*; we keep the oversampled eye trace for the next step–correction of the reference frame by interpolation–but a physically meaningful eye trace would be divided by *k*.

#### Removing outliers in lag bias estimation

Using we can look at the distribution of locations in a given target frame to which the reference strips have matched. Given the frame rate of the imaging system and estimates of maximum eye movement velocities [26], it is possible to tell whether the distributions of eye position for a given frame, e.g. $\hat{s_{x}}\left(f_{0},r\right)$ and $\hat{s_{x}}\left(f_{0},r\right)$, are reasonable. A number of approaches for detecting outliers are possible; we employed Tukey’s fences. Letting *Q*3 and *Q*1 represent the third and first quartiles, respectively, with interquartile range IQR = *Q*3 − *Q*1, we discarded any data outside the range [*Q*1 − 1.5 ⋅ *IQR*, *Q*3 + 1.5 ⋅ IQR].

#### Using estimated eye movement traces to correct the reference image

The reference image *I*_Γ_ is corrected for eye movements as follows. First, a set of uniformly-spaced coordinates spanning the width and height of the reference image is defined:

$$\begin{array}{c} \begin{array}{c} \Gamma_{0}=\{\left(X_{0},Y_{0}\right),\left(X_{1},Y_{0}\right),\ldots \left(X_{C-1},Y_{0}\right),\\ \left(X_{0},Y_{1}\right),\left(X_{1},Y_{1}\right),\ldots \left(X_{C-1},Y_{1}\right),\\ \ldots \\ \left(X_{0},Y_{R-1}\right),\ldots \left(X_{C-1},Y_{R-1}\right)\} \end{array} \end{array}$$ (6)

The coordinate set Γ_0_ can be thought of as the retinal coordinates that would be imaged by a scanning beam in the absence of eye motion. It is the coordinate set into which the motion-affected image will be interpolated. Next, using our reconstruction of the eye’s movements, ${\hat{x}}_{t}$ and ${\hat{y}}_{t}$, we define the retinal coordinates at which the pixels were acquired, Γ:

$$\begin{array}{c} \begin{array}{c} \Gamma =\{\left(X_{0}+{\hat{x}}_{0},Y_{0}+{\hat{y}}_{0}\right),\left(X_{1}+{\hat{x}}_{0},Y_{0}+{\hat{y}}_{0}\right),\ldots \left(X_{C-1}+{\hat{x}}_{0},Y_{0}+{\hat{y}}_{0}\right),\\ \left(X_{0}+{\hat{x}}_{1},Y_{1}+{\hat{y}}_{1}\right),\left(X_{1}+{\hat{x}}_{1},Y_{1}+{\hat{y}}_{1}\right),\ldots \left(X_{C-1}+{\hat{x}}_{1},Y_{1}+{\hat{y}}_{1}\right),\\ \ldots \\ \left(X_{0}+{\hat{x}}_{R-1},Y_{R-1}+{\hat{y}}_{R-1}\right),\ldots \left(X_{C-1}+{\hat{x}}_{R-1},Y_{R-1}+{\hat{y}}_{R-1}\right)\} \end{array} \end{array}$$ (7)

The final step is correcting the eye movement artifacts in the reference image. Given the two coordinate sets Γ and Γ_0_, a two-dimensional interpolation algorithm *M* is employed to interpolate intensities at Γ_0_ using the observed pixel intensities *I*_Γ_ found at Γ:

$$\begin{array}{c} I_{\Gamma_{0}}=M\left(I_{\Gamma },\Gamma ,\Gamma_{0}\right) \end{array}$$ (8)

For the results presented below, we chose a two-dimensional cubic interpolation using triangulation followed by Bezier polynomial interpolation in each triangle using the Clough-Tocher algorithm [33]. This algorithm was selected because it insures that there are no discontinuities in the resulting image, though there were no visible differences between the result of this approach and either a simpler two-dimensional linear interpolation or a nearest-neighbor interpolation similar to that employed by Bedggood and Metha [23]. $I_{\Gamma_{0}}$ was then used as a reference frame for a second round of the strip-registration steps described above, in place of *I*_Γ_.

### Validation of image reconstruction

#### Validation of image reconstruction using synthetic images

In the case of synthetic images, an exact record of simulated eye movements and a motion-free version of the reference image were available for direct comparison with lag-bias reconstructions. In order to validate the estimate of eye movements, *R*^2^ values for the fits were calculated using records of eye motion *x*_*t*_ and *y*_*t*_ with average positions of $\bar{x_{t}}$ and $\bar{y_{t}}$ and reconstructions ${\hat{x}}_{t}$ and ${\hat{y}}_{t}$:

$$R_{x}^{2}=1-\frac{\sum_{t}{\left(x_{t}-{\hat{x}}_{t}\right)}^{2}}{\sum_{t}{\left(x_{t}-\bar{x_{t}}\right)}^{2}}$$ (9)

$$R_{y}^{2}=1-\frac{\sum_{t}{\left(y_{t}-{\hat{y}}_{t}\right)}^{2}}{\sum_{t}{\left(y_{t}-\bar{y_{t}}\right)}^{2}}$$ (10)

$R_{x}^{2}$ and $R_{y}^{2}$ describe the fraction of variance in eye position accounted for by the reconstructed eye movement traces.

To validate the reconstruction of the reference frame, whole-image cross-correlations between the motion-free reference and each of the uncorrected and corrected reference images were performed. These were performed using a formula similar to Eq 3, while normalizing for the size of overlap between the shifted images [1].

#### Validation of image reconstruction using AO-SLO images

In the case of retinal images acquired with the AO-SLO, neither eye movement traces nor a motion-free image of the retina were available for direct comparison with the lag-bias reconstruction. As such, an indirect method of validation was devised. From series of 100 AO-SLO images, multiple frames were selected as references for strip-based registration, and each of these was corrected using the approach described above. Pairs of uncorrected images were compared with whole-image cross-correlation, and the corresponding corrected images were compared the same way. Because the number of pixels used in the calculation of image correlation *ρ* (i.e., peak cross-correlation) was very high (> 16, 000), we believe that the probability of a chance improvement in image correlation is negligible, and that improvements in image correlation could only be possible if correspondence between the images and object were improved.

We employed a second method to validate the motion-correction algorithm, based on direct measurement of motion artifacts in the reference image before and after correction. The method, proposed by Salmon et al. [21], detects motion artifacts by measuring shear in the image using DFT (F). For an image *I* of a hexagonal mosaic of round objects with some topological disarray, the spatial power spectrum $F{\left(I\right)}^{2}$ is a radially symmetric ring, sometimes referred to as Yellott’s ring [34]. In the presence of shear in the image, due to eye movements with a component parallel to the fast scanner’s axis, spatial frequencies in the direction of the shear become lower, which leads to compression of the power spectrum ring along that dimension and an elliptical power spectrum.

In order to detect shear, we thresholded the power spectrum at 25% of its mean value, and then recorded the y-coordinate of the maximum value in each of its columns, as well as the x-coordinate for each column. Next, a linear regression was applied to these coordinates and the coefficient of determination (*R*^2^) was determined. This process was applied to 50-pixel wide strips of the image, in order to prevent washout of the effect due to complementary eye movements within the same image. For strips with negligible shear and correspondingly circular power spectra, we expected low values of *R*^2^. For strips with shear and visibly elliptical power spectra, we expected high values of *R*^2^. To validate the motion-correction algorithm, we computed strip-wise values of *R*^2^ before and after correction.

#### Application of strip-registration and motion correction to other imaging modalities

To test the generality of our strip-based method, we tested it on images acquired with other modalities. We acquired non-confocal images with the AO-SLO, namely the split-detector and dark field channels, concurrently with confocal images. The confocal images were used to register the non-confocal images in a *dual registration* approach [13].

In addition, we tested the algorithm on AO-OCT volumes, in both the lateral and axial dimensions. First, areal projections of the cone mosaic (IS/OS and COST layers) were used to perform strip-based lateral registration and lag-bias reconstruction. Next, cross-sectional projections were used to perform strip-based axial registration and the resulting lag biases were used to correct axial motion. The resulting motion-free voumetric images were used to create maps of cone inner segment and outer segment lengths, and to project other layers such as the Henle fiber layer (HFL) and retinal pigment epithelium (RPE).

## Results

The proposed reconstruction algorithm was tested in two ways. First, it was applied to synthetic images (phantoms) of the cone photoreceptor mosaic in which movement artifacts were simulated by virtually moving the phantom while sampling it with a raster pattern. The resulting reconstructions were compared with the motion-free version. Second, it was applied to retinal images acquired using our AO-SLO and AO-OCT systems. Because motion-free images of these retina were not available, the reconstructions were compared using indirect methods. After describing these validation results, we show some of the resulting improvements AO-SLO and AO-OCT images and analyses.

### Intraframe motion correction using synthetic images with motion artifacts

**Fig 5:**
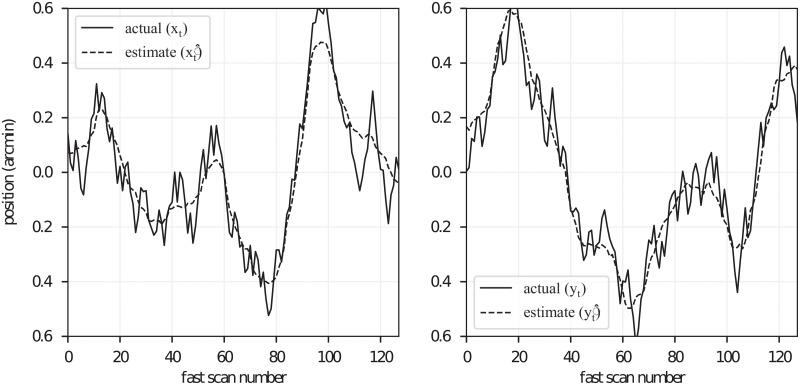
Estimation of simulated motion from strip-registration lag biases. Traces of the simulated eye position are plotted with solid lines, separately for horizontal (left) and vertical (right) components. The lag bias estimates ${\hat{x}}_{{}_{t}}$ and ${\hat{y}}_{{}_{t}}$ are plotted with dashed lines. Estimates bear qualitative similarity to the simulated movement trace, consistent with high goodnesses of fit to the simulated movement trace ($R_{x}^{2}=0.96$ and $R_{y}^{2}=0.96$).

Fig 5 (solid black lines) shows x- and y-components of the eye movements simulated during acquisition of the synthetic reference frame. The motion-free image, two-dimensional movement plot, and corrupted reference frame are shown in Fig 1. Similar–but uncorrelated–eye movements were simulated for acquisition of the remaining 99 images in the 100-image series. Resulting cross-correlation lag biases were used to estimate the simulated eye movements, and these are shown in Fig 5 (dashed black lines). The simulated movements and estimates of movement bear visible correspondence, with *R*^2^ values of 0.96 for both x and y fits.

**Fig 6:**
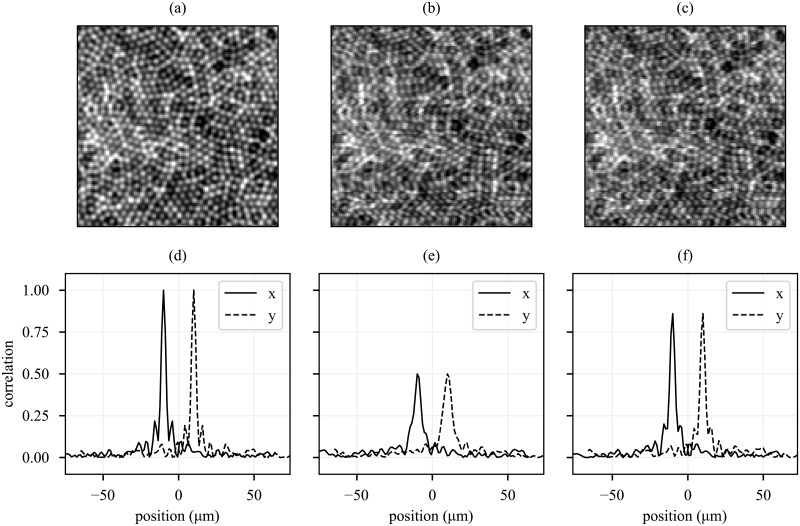
Removal of motion artifacts from reference image. After reconstructing reference frame eye movements from lag biases, the reference frame is interpolated from its natural coordinates into a set of motion-free coordinates. The original object is shown in (a). The image used as a reference for strip-based registration is shown in (b). The motion-corrected reference is shown in (c). The shear, compression, and expansion artifacts in (b) are visibly reduced in (c). In order to demonstrate better correspondence of the corrected reference to the object, horizontal and vertical profiles of whole-image cross-correlations with (a) are shown. Horizontal and vertical profiles are offset for clarity. (d) shows the autocorrelation of (a), with characteristic central peak and side lobes due to the regularly spaced cones. (e) and (f) show the whole-image cross-correlations between (b) and (a) and between (c) and (a), respectively. Correlation of the corrected image with the object is significantly higher, with the residual mismatch between the latter two limited by finite oversampling of images during cross-correlation.

Fig 6 shows the simulated object (a), image with simulated motion artifacts (b), and lag bias reconstruction (c). Shear, compression, and expansion artifacts are evident in (b) and visibly reduced in (c). Fidelity of these images was quantified by performing a two-dimensional whole-image cross-correlation of each with the object (a). The autocorrelation of the object is shown in (d), with a peak value of 1.0 and side lobes characteristic of the object’s periodic structure. Cross-correlation of the motion-corrupted image (b) and object (a) is plotted in (e). Motion artifacts clearly reduce the correlation of the images, with a peak correlation of 0.5 and loss of the side lobes. Cross-correlation of the lag-bias reconstruction (c) and object (a) is plotted in (f), with a peak correlation of 0.86 and recovery of the side lobes.

Simulation of the retinal image and eye movements permitted direct comparison of both the eye movement estimate and reconstruction with the known simulated eye movements and object. In both instances the algorithm performed well, as evidenced by the high *R*^2^ values and correlation recovery, respectively.

### Comparison with a previous approach

**Fig 7:**
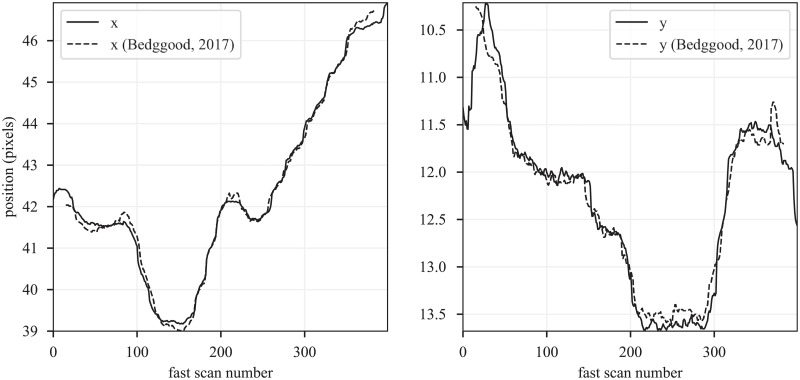
Comparison with previously published approach. The method proposed here and the method proposed by Bedggood and Metha, 2017 are slightly different. Our method does not utilize rigid body registration of whole frames prior to strip registration. As such, we may expect minor differences in the reconstructed eye movement traces, owed in part to the higher probability for strips to be misplaced in our approach. In order to quantify this error, we compared eye motion reconstructions using the Bedggood and Metha method with our own. The resulting pairs of x and y movement traces are shown on the left and right. The reconstructed traces from the two methods are qualitatively similar, and have *R*^2^ values of 1.0 and 0.97, respectively.

A distinction between the method presented here and that presented in Bedggood, 2017 [23] is that in our approach, rigid body pre-registration of the frames was not employed. Instead, by registering reference strips to entire target frames, both the rigid body lag and strip lag are determined together. Without the benefit of rigid-body pre-registration, our approach my be more susceptible to spurious matching of strips to incorrect parts of the target frame. We sought to remove these mismatches using outlier detection. Due to these differences, small amounts of error may be expected. In order to verify that the error was small, we compared the reconstructed eye traces generated by their approach and our approach using images published as a supplement to their paper [23]. The results, shown in Fig 7, indicate very close correspondence between the resulting movement traces. The *R*^2^ values were 1.0 and 0.97 for the x and y traces, respectively.

### Intraframe motion correction using AO-SLO images of the cone mosaic

**Fig 8:**
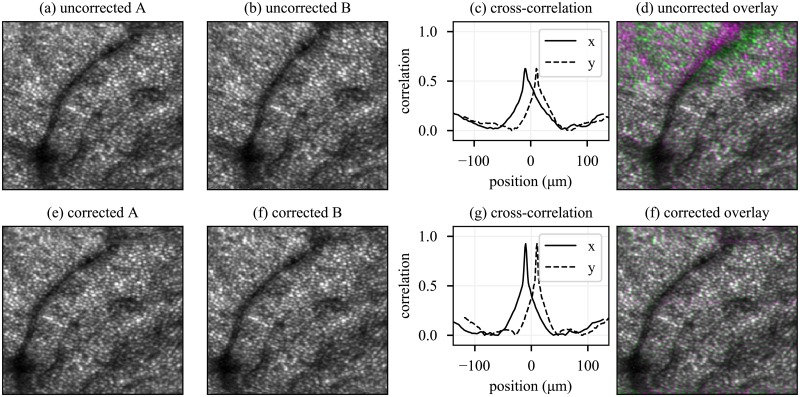
Removal of motion artifacts from real AO-SLO images. (a) and (b) show an average of 100 AO-SLO frames strip-registered to two separate reference frames; (e) and (f) show the motion-corrected versions of (a) and (b), respectively; (c) and (g) show the cross-correlations *a* ⋆ *b* and *e* ⋆ *f*, respectively. Clearly, smearing of the cones in (a) and (b) has reduced the cross-correlations between them, and after motion-correction of the references, the cross-correlation in both x- and y- direction become sharper and its value increases. (d) and (h) show a pseudocolor overlay of (a,b) and (e,f), respectively.

While direct comparison of AO-SLO image reconstructions with eye movements or object structure were not possible, an indirect method of comparison was employed. Two reference images were selected from a series of 100. Frames were intentionally selected which exhibited visible differences due to eye movements. The strip-based registration algorithm was used to register the remaining 99 frames to each reference. The resulting average images are shown in Fig 8(a) and 8(b). Whole-image cross-correlation of the resulting averages (a) and (b) is plotted in panel (c), showing poor correlation between the images. The mismatch between the images is visible in the pseudocolor overlay of (a) and (b), which is shown in (d). The reference images used to generate averages in Fig 8(a) and 8(b) were independently corrected using their separate strip-registration lag biases. The resulting reconstructions are shown in (e) and (f), and bear better visual correspondence than (a) and (b). Whole-image cross-correlation of (e) and (f) reveals a significantly higher peak value (0.93), with mismatches barely visible in the pseudocolor overlay shown in (g). Our interpretation of the improved correlation between the two registered averages is that it is highly unlikely to have occurred by chance, and that it is most easily explained by presuming that both corrected images represent the object structure more faithfully than their uncorrected antecedents.

**Fig 9:**
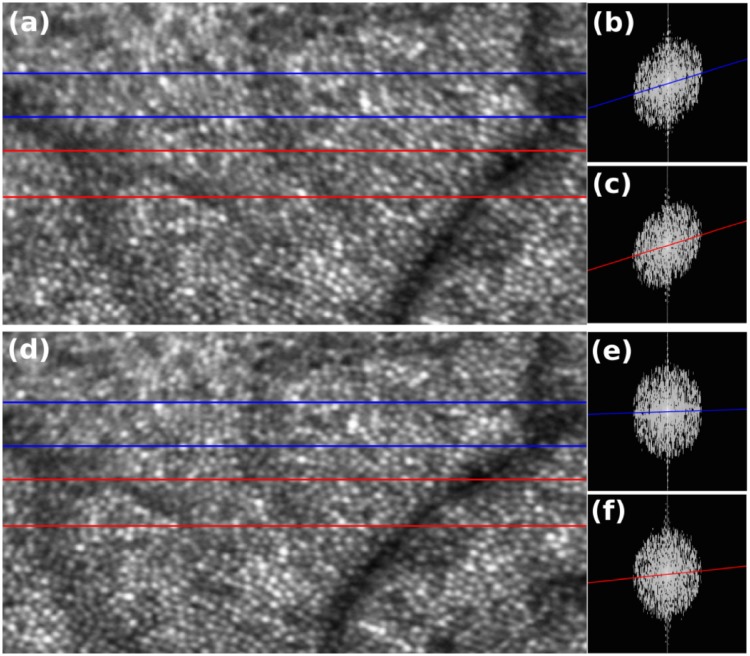
Motion analysis of the averaged AO-SLO image before and after motion correction. (a) Averaged AO-SLO image before motion correction; (b) and (c) Logarithmic scale DFT of the area outlined in blue and red strips in panel (a). Distortion analysis based on the coefficient of determination (*R*^2^) obtained from the linear regression of the maxima across columns shows *R*^2^ values of 0.327 and 0.276 for the area outlined in blue and red, respectively. (d) Averaged AO-SLO image after motion correction algorithm; (e) and (f) logarithmic scale DFT of the area outlined in blue and red strips in panel (d). The proposed motion correction algorithm reduced the distortion of the averaged image where the *R*^2^ values reduced to 0.004 and 0.042 for the area outlined in blue and red in panel (d), respectively.

Performance of the reconstruction algorithm on real AOSLO images was assessed in a second, independent way. The DFT of horizontal strips of images of the cone mosaic were thresholded and fitted with a linear regression, permitting computation of coefficients of determination (*R*^2^). A large value of *R*^2^ indicates a shearing artifact in the strip, whereas values close to zero indicate absence of such artifacts. Fig 9 shows the result of this assessment. A strip-registered average without intraframe motion correction is shown in (a), with two strips indicated with blue and red boxes. Log-scale two-dimensional DFTs of those two strips are shown in (b) and (c), respectively, with regression lines superimposed. Corresponding images and plots, after lag bias reconstruction, are shown in (d), (e), and (f), respectively. The *R*^2^ values prior to correction were 0.2 and 0.3, for the blue and red regions, respectively. After reconstruction, these values fell to 0.004 and 0.042, respectively, indicating substantial removal of shearing artifacts.

**Fig 10:**
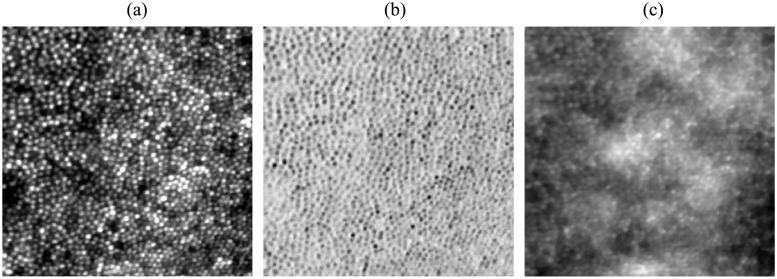
Strip-based registration of non-confocal AO-SLO channels. (a) confocal, (b) split-detector, and (c) dark-field at 1° temporal to the fovea. The high-contrast image provided by the confocal channel permits strip-registration and motion correction of images in the other channels, since the images are acquired concurrently.

The AO-SLO system is capable of acquiring concurrent confocal, split-detector, and dark-field images. Examples, collected at 1° on a normal subject, are shown in Fig 10. We have demonstrated the effectiveness of the lag bias method on AO-SLO confocal images (Figs 8 and 9), and since confocal images are acquired concurrently with split-detector and dark-field images, the latter can be corrected using the same eye movement record. The algorithm was not tested on split-detector or dark-field images directly.

### Intraframe motion correction using volumetric AO-OCT images of the retina

**Fig 11:**
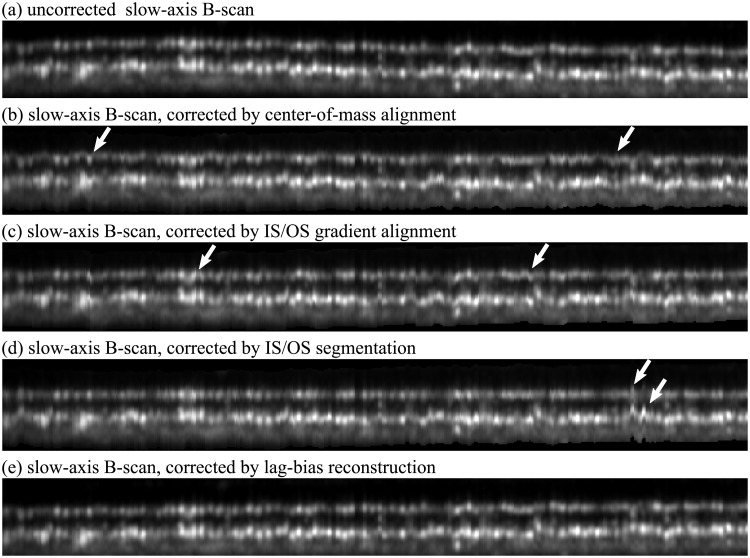
Correcting axial motion in AO-OCT volumetric images. Slow-axis B-scans of the IS/OS and COST layers. The uncorrected scan is shown in (a), with visible axial eye motion. *En face* projection and other visualization and analysis techniques require correction of axial warp, and commonly employed techniques include alignment of B-scans by center of mass, gradient-based edge detection, and maxima segmentation, shown in (b), (c), and (d), respectively. While the outer segments of cones in any region of interest are similar, they are not identical, and the reflective surfaces forming their boundaries are axially staggered with respect to one another. A consequence of this staggering is that aligning by these methods results in artifacts (white arrows) as well as alteration in the apparent flatness of the surfaces. The latter artifact is especially evident in (d), where the IS/OS is artificially flattened while the COST is artificially made rougher. Lag-bias reconstruction (e) avoids these artifacts, and is presumed to yield a more faithful representation of the roughness of these surfaces.

Strip-registration and lag-bias reconstruction were also successfully applied to AO-OCT images. In the first, lateral reconstruction step, the results were very similar to AO-SLO improvements shown above. The resulting volume may still possess artifacts of axial motion, manifesting as axial warp, as shown in Fig 11(a). A number of approaches have been proposed for flattening OCT volumes, such as alignment by center of mass (b), gradient-based edge-detection (c), and axial peak segmentation (d). While these approaches can be useful for *en face* projection of the outer segment slab, they all result in artifacts (white arrows). Moreover, each of these approaches alters the axial position of A-scans in ways that may misrepresent the cellular structure. We have employed approaches (b), (c), and (d) in previous projects, and found that artifacts can be removed via various *ad hoc* fixes, such as low-pass filtering of segmentation traces, or empirical tuning of graph cut segmentation [35] parameters, but lag-bias correction is possibly a more general approach to the problem.

**Fig 12:**
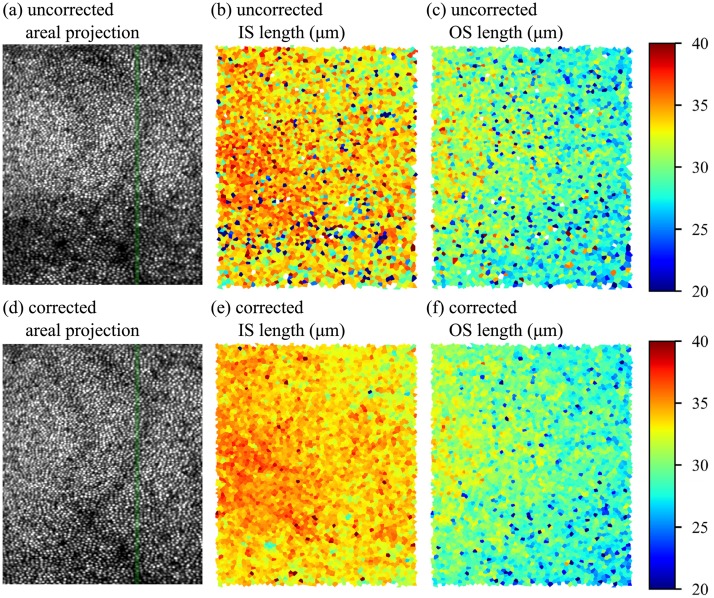
Axial lag bias reconstruction improves accuracy of cellular morphometry. AO-OCT volumes permit many sorts of quantitative cellular morphological measurements. The curvature of the cone bands results in variations in *en face* projections of the cone mosaic along the slow (vertical) axis of the image, as shown in (a) and (d). The green line indicates the location of the slow-axis B-scans shown in Fig 11(a) and 11(e), respectively. Automated three-dimensional segmentation of inner (IS) and outer segments (OS) permits visualization of their lengths. Uncorrected IS and OS lengths are shown in (b) and (c), while corrected maps are shown in (e) and (f). While a ground-truth comparison is not possible, the corrected maps appear to suffer from fewer errors and bear better similarity to the smooth appearance of the layers in OCT B-scans and other morphometric studies [38]. The IS map (b) and (e), which requires localization of the relatively dim ELM, benefits especially from careful correction.

**Fig 13:**
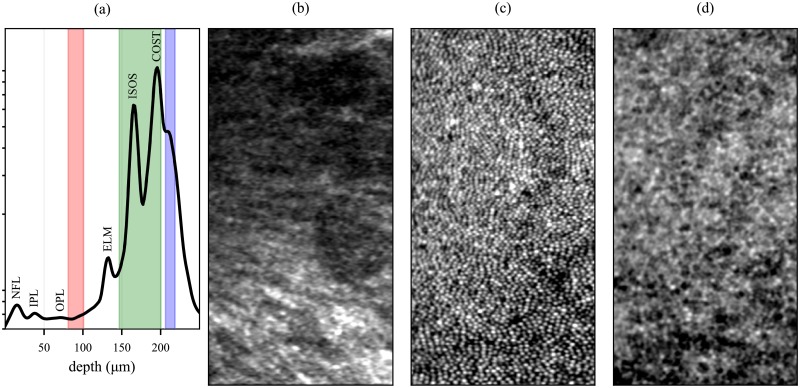
Removal of motion artifacts from AO-OCT images. Averaging the AO-OCT volume in the two lateral dimensions produces a longitudinal reflectance profile, shown (a) in log scale. The labeled peaks correspond to the nerve fiber layer (NFL), inner plexiform layer (IPL), outer plexiform layer (OPL), external limiting membrane (ELM), inner-outer segment junction (ISOS), and outer segment tips (COST). The axial extents of the Henle fiber layer (HFL), cone outer segments (COS), and retinal pigment epithelium (RPE) are depicted on the plot with red, green, and blue shaded boxes. By extracting and averaging together corresponding depths of interest from the motion-corrected volumetric image, projections of these layers can be produced, shown in (b), (c), and (d), respectively. Some of the variation in brightness of HFL is likely due to shadows cast by overlying blood vessels; these can be observed in the much brighter COS mosaic as well. Other factors may be segmentation errors and directional effects [39, 40]. Each pixel in the images consists of an average of between 90 and 135 separate measurements. Images were centered 0.5° temporal to the fovea, subtending 1° and 0.5° in the vertical and horizontal dimensions, respectively.

Recent studies of the anatomical origins and properties of the outer retinal bands have shown that they are broader than would be predicted by the thin reflectors hypothesized to exist at IS/OS and COST [36], and that one of the factors in their broader appearance may be the axial staggering of surfaces among neighboring cells combined with lateral blur [32, 37]. Table 1 shows how estimates of this axial staggering may depend upon the chosen method of flattening. Because the lag-bias approach makes no assumptions about which aspect of the reflectors (gradient, intensity, or center of mass) is the most germane, we propose that it provides the most faithful representation of the structure. The benefits of motion correction can be seen in futher morphometric studies of the photoreceptors, such as the maps of inner and outer segment length shown in Fig 12 and projection of other retinal layers (Fig 13).

**Table 1:** Roughness of outer retinal surfaces depends on axial dewarping strategy.

	IS/OS roughness	COST roughness
	(*μm* RMS)	(*μm* RMS)
uncorrected	3.7	4.2
center of mass alignment	11.3	7.3
gradient segmentation	1.4	2.5
peak segmentation	0.0	2.6
lag bias reconstruction	2.2	2.8

Axial motion correction of AO-OCT volumes also permits better segmentation of various layers of the volumetric image. A longitudinal reference profile (LRP) of the corrected volume is shown in Fig 13(a), with peaks attributed to retinal layers labeled. From the corrected volume, three *en face* projections were generated: the Henle fiber layer (HFL), cone outer segment (COS), and retinal pigment epithelium (RPE), shown in (b), (c), and (c), respectively. The radial, linear structure of HFL is visible in (b), while the dark nuclei of RPE cells is visible in (d).

## Discussion

Lag-bias correction of intraframe motion represents a technique for correcting lateral and axial eye movement artifacts in raster-scanned retinal images, be they two-dimensional AO-SLO images or three-dimensional AO-OCT images.

### Potential applications for lag-bias reconstruction

More faithful representations of the retina would improve the accuracy of morphological measurements of retinal features. Thus improved accuracy could be leveraged to detect smaller changes in the size of retinal cells and vasculature, establishment of more precise norms, or earlier detection of deviations from norms. Artifacts of fixational drift (∼0.5°/*s*) among lines acquired with a typical (16 kHz, with 1 *μm* sampling of the retina) AO-SLO are very small, resulting in shears, compressions, or expansions of about 0.1%. The consequent loss in morphometric precision is likely negligible for most current applications, though further improvements in resolution and speed [14] may increase our demands for precision. Moreover, at the relatively lower line rates of the fastest scanning AO-OCT systems (up to 2 kHz for a field of view comparable to that of the AO-SLO [41]), or when considering microsaccades in AO-SLO images, the retina may move by more than one micron during each fast scan, which effectively doubles or zeros the apparent size of an object in the cases of pure expansion or compression, respectively. In the case of shear, where the retina moves parallel to the fast scanner, the apparent area of an object is not affected, though measurements of its length or diameter are. Geometric properties other than size, such as shape, orientation, and curvature are affected by all eye movements.

Some functional AO applications require tracking of cellular or other microscopic features over time. In principle, to monitor these changes, all images could be registered to a single reference image, ignoring distortions of the features. In practice, registration of images collected over long spans of time can be complicated by changes in retinal appearance [3, 7, 11, 16] and normal variation in the eye’s optical properties and/or performance of the AO system. Lag-bias reconstruction might prove useful in these cases by permitting application of rigid body techniques to true images.

From the first AO images of the cone mosaic, the power spectrum has been used to visualize and quantify the packing density of the cones [42]. Since then, photoreceptor density has been used extensively to characterize the healthy retina as well as retinal disease [43–48]. The same approach has been successfully applied in RPE characterization [17] and choriocapillaris imaging [49] as well. As can be observed in Fig 9, eye movements create distortions in the power spectrum which reduce the accuracy of the resulting estimates of spatial frequencies. Lag-bias reconstruction could thus be employed to improve frequency-domain estimates of the density of periodic structures in the retina.

In addition to providing a way to correct motion artifacts in the retina, lag-biases provide a method for verifying other methods of correcting motion, whether through active tracking or post-processing. The motivating principle, that lag-biases will approach zero when the reference image is free from motion artifacts, implies that quantities such as the mean absolute bias or bias RMS could be used as figures of merit for other tracking and correction methods. Because this approach permits a reconstruction of the eye movement trace (Fig 5), residual movement artifacts can be characterized and quantified in numerous ways.

Cross-modal registration is useful for studying the relationships between different aspects of retinal structure and/or function. Registering, for instance, OCT angiograms with structural intensity images of the neural retina could be very useful for determining whether hypoperfusion may be correlated with degeneration of photoreceptors or other retinal neurons. Cross-modal registration is complicated by a number of factors, such as differences in sampling density, linearity of the fast scanner, and orientation of the scanners. If, however, images from both modalities are corrected using lag-bias reconstruction, the registration problem simplifies to the affine subset of rotation, translation, and scaling.

Lastly, by permitting removal of axial motion artifacts in OCT B-scans, lag-bias reconstruction permits a more faithful measurement of the retina’s axial structure. This improvement could be leveraged to improve morphometric measurements [32, 37, 38] as well as measurements of retinal function which depend on quantifying changes in axial morphology [11, 16, 50–52].

### Limitations of lag-bias reconstruction

The proposed method has important limitations as well. Because motion-free images of the relevant structures are typically not available, ground truth validation of reconstructions cannot be made. Moreover, this method does not address deformations due to biases introduced by the imaging system. For instance, if the axes of the two scanners are not perpendicular or if the scanners (in OCT) are not conjugated with the eyeball’s center of curvature, the resulting images are deformed in ways that would not be revealed by our method.

The proposed method appears to work in the case of stochastic, radially symetrical directions of eye movements. Investigators have shown that some fixators have “idiosyncratic” eye movements, in which one direction of drift predominates [53, 54]. If the idiosyncratic drift is not compensated with corrective microsaccades, it would be undetectable using our approach, since all strips in the reference will be shifted by the same amount for each target frame. It is possible that the bulk shifts could be used to infer intraframe drift, but this possibility was not explored. On the other hand, if the idiosyncratic drift is accompanied by corrective microsaccades, and if the tissue can be reliably imaged during the microsaccades, then the intraframe motion can be corrected. For AO-SLO images with a spatial sampling of 1.0 *μm* per pixel and a line rate of 16 KHz, microsaccades with a velocity of 30 deg/s would result in a shear of 0.56 pixels per line. With sufficient image contrast and sufficient overlap between reference and target frames, both the drift and corrective saccade should be measured, and the resulting lag biases could be used to correct a reference frame even if it contains a microsaccade. An example has been included in the software published with this manuscript.

Many quantitative studies of retinal morphology depend on the *topology* of features in the image, as opposed to their geometric properties. Examples of topological properties of the cone mosaic are the *number of neighbors* possessed by a cone and the overlap of features in axially separated retinal layers. These properties have been used to measure cone loss in genetic color deficiencies [55] and to describe the way in which photoreceptors are distributed with respect to RPE cells [17]. Neither would likely be improved by the correction of motion artifacts.

### Parameter choices

The whole algorithm, including both the strip registration steps and the lag-bias reconstruction, has a number of free parameters. While we did not systematically test the effects of these on the resulting motion correction, we found a set of values that worked for our synthetic and AO-SLO data sets. For data acquired using a substantively different modality (e.g., AO-OCT), different values were optimal.

One free parameter is *strip overlap*. We chose to increment *r* by one for each strip, producing a set of mostly overlapping strips equal in number to *R*, the number of rows in the image. We selected this value in order to have the densest possible estimate of eye movement, but it came at the price of computation time. Sparser estimates of eye movement using disjoint strips may be sufficient to remove the bulk of motion artifacts. To produce disjoint strips, the same approach can be employed while incrementing by *W* instead of one.

The shape of the window may be another relevant parameter. We chose a rectangular window (see Eq 1), such that the rows constituting the strip carry equal weights in the cross-correlation. Non-rectangular windows (e.g., a Gaussian window with suitable width *W*: *ω*_*r*_(*p*) = exp[−(*p* − *r*)^2^/(2 *W*^2^)]) may be an alternative with some advantages, though we did not explore this idea.

Another parameter was window width. We used a windows of 9 and 7 pixels, for AO-SLO and AO-OCT, respectively, for the images shown above. We found strips as narrow as 1 pixel (AO-OCT) and as wide as 15 pixels (AO-SLO) were effective. Increasing strip width tends to smooth the resulting eye movement estimates and also to reduce the number of outliers (mismatched strips), presumably at the expense of accuracy.

Increasing the oversampling factor *k* could be useful, provided that the system is limited by oversampling rather than optical factors. We chose a value of three, simply because higher values of *k* were impractical due to computational demands.

### Possible sources of error

As shown in Figs 5 and 6, the lag-bias approach does not generate perfect reconstructions of eye movements or object structure. Here we briefly consider some of the sources of error.

One likely source of error is the finite oversampling of images. In order to achieve a precision of 0.33 pixels, we oversampled images by a factor of 3 prior to strip-registration. While the two-dimensional interpolation used to correct motion artifacts in the reference image has arbitrarily high precision, the finitely oversampled images used to produce registered averages will–even in the best case scenario–have errors at higher spatial frequencies. These would manifest in a reduction in image contrast and subsequent reductions in correlation between images (Fig 6).

Error due to finite sampling would impact some applications of lag-bias reconstruction, but not others. If the geometric relationship between features in the image is the crucial dependent variable, this source of error may have minimal impact. However, where the reflectivity of retinal features is of great importance, finite sampling may impose a limit on precision.

Whereas we chose our oversampling factor somewhat arbitrarily, an exact value could be calculated for *k*, using the modulation transfer function (MTF) of the system in conjunction with the Nyquist-Shannon theorem, such that the optical properties of the system (and eye) would impose a bottleneck on modulation transfer, rather than the oversampling factor. We do not know whether our images were limited by optical properties or digitization.

A second source of error is sampling error, due to the finite number of target image strips used to estimate the lag biases. Sampling error may impact estimates of eye movement because the lag biases ${\hat{x}}_{t}$ and ${\hat{y}}_{t}$ are averages over a finite number (*F*) of frames. This error likely limits both estimation of eye movement and reconstruction of motion-free references. Using our simulated images, we determined that an *R*^2^ value of 0.95 was achieved by registering reference strips to between 25 and 60 frames. For real AO-SLO images, cross-correlation of two corrected images was 0.93 using 100 frames (see Fig 6). Using 25 and 50 images resulted in cross-correlations of approximately 0.8 and 0.9, respectively. The optimal number of frames required would depend on the scientific or qualitative demands of the application.

## Conclusion

The approach demonstrated here shows that eye movement artifacts in reference frames reveal themselves in the statistics of stripwise cross-correlation dewarping. While correction of these artifacts may be of little consequence for wide-field imaging of the retina, with AO it may permit more precise measurements of the spatial distribution of cells or vascular morphology and, in particular, facilitate detection of the changes accompanying retinal diseases.
